# Musculature adaption in patients with lumbosacral transitional vertebrae: a matched-pair analysis of 46 patients

**DOI:** 10.1007/s00256-021-03722-x

**Published:** 2021-02-03

**Authors:** Luis Becker, Katharina Ziegeler, Torsten Diekhoff, Yannick Palmowski, Matthias Pumberger, Friederike Schömig

**Affiliations:** 1 Center for Musculoskeletal Surgery, Charité – University Medicine Berlin, Charitéplatz 1, 10117 Berlin, Germany; 2 Department of Radiology, Charité – University Medicine Berlin, Berlin, Germany

**Keywords:** Transitional, Adaptation, Atrophy, Asymmetry

## Abstract

**Objective:**

Even though lumbosacral transitional vertebrae (LSTV) are one of the most common congenital anomalies of the spine, their effect on surrounding soft tissues is not well-studied. We therefore aimed at analyzing the association between LSTV and changes in volume, mass, symmetry, and degeneration of lumbar and trunk muscles.

**Materials and methods:**

Abdomen–pelvis CT scans were analyzed in patients with LSTV and a matched control group. LSTV were classified according to the Castellvi classification. Muscles were segmented from the remaining soft tissue and their cross-sectional area and volume were examined at five defined levels. Threshold segmentation was used to differentiate between muscle fibers and fat tissue. Matched pairs were compared using Wilcoxon rank sum tests. For comparison of categorical data, chi-squared tests were performed and for associations between the degree of fusion and muscle size and degeneration, Spearman’s correlation coefficients were calculated. Inter- and intrarater reliabilities were evaluated by computing intraclass correlation coefficients.

**Results:**

Forty-six patients with LSTV and 46 controls were included. Muscle volume of the paraspinal and trunk muscles was significantly lower (707.0 cm^3^ vs. 809.7 cm^3^, *p* < 0.001) and fatty muscle changes were significantly increased in all but the caudal paravertebral muscles of LSTV patients (M. psoas *p* < 0.04, M. quadratus lumborum *p* < 0.001, paravertebral muscles *p* = 0.011, M. rectus abdominis *p* < 0.001, M. obliquus abdominis *p* < 0.001). Correlations between the degree of Castellvi classification and muscle volume were significant (*p* = 0.001).

**Conclusion:**

LSTV are associated with a reduction in muscle volume and an increase in muscle degeneration of both lumbar and trunk muscles.

## Introduction

Lumbosacral transitional vertebrae (LSTV) are one of the most common congenital anomalies of the spine, with a reported prevalence of 4–36%, and are defined as either a sacralization of the lowest lumbar segment or a lumbarization of the superior sacral segment [1, 2]. Castellvi et al. introduced a radiographic classification system for LSTV in 1984, defining four different types of LSTV: Type I includes unilateral (Ia) or bilateral (Ib) dysplastic transverse processes; type II is an incomplete unilateral (IIa) or bilateral (IIb) lumbarization or sacralization with an enlarged transverse process with a diarthrodial joint between itself and the sacrum; type III is a unilateral (IIIa) or bilateral (IIIb) lumbarization or sacralization with complete osseous fusion of the transverse process to the sacrum; and type IV is defined as a unilateral type II transition with a type III transition on the contralateral side [3]. Diagnosis of LSTV classically includes lateral and Ferguson radiographs but characterization of LSTV is best performed by computed tomography (CT) [1].

Despite some controversies in the literature, there appears to be an association between LSTV and the occurrence of low back pain (LBP) caused by various etiologies, such as disc or spinal canal pathology at the level above transition, degeneration of the anomalous articulation, facet joint arthrosis, and stenosis secondary to a broadened transverse process [1]. However, other studies reported no correlation between LBP and LSTV and no increased incidence of structural pathologies in patients with LSTV [4, 5].

Core trunk and low back muscle atrophy has been shown to be associated with LBP and, with less strong evidence, with degenerative disc disease and spinal stenosis [6–9]. As the posterior muscles of the lumbar spine provide stability to the lumbar vertebral segments and control movement of the lumbar spine, muscular integrity plays an important role in the maintenance of global spinal alignment [10, 11]. Previous studies have shown that atrophy of paraspinal muscles may lead to altered thoracic kyphosis, lumbar lordosis, and sacral–vertebral angles both in adults with and without degenerative spinal disease [12–14]. Due to this important role trunk and lumbar muscles play in spinal integrity and pain development, physiotherapy has been shown to significantly improve functionality and positively alter the course of LBP [15].

To determine whether the occurrence of LSTV is associated with changes in the psoas muscle, quadratus lumborum muscle, rectus abdominis muscle, abdominal oblique muscle, and paraspinal muscles, we analyzed muscle volume, mass, symmetry, and degeneration at five defined levels of CT scans of patients with and without LSTV using the image processing software Amira.

## Materials and methods

### Patients and ethics approval

A retrospective matched-pair analysis of the abdomen–pelvis CT scans was performed. The study was approved by the local ethics board (ethics proposal number EA1/300/19). Included patients underwent abdomen–pelvis CT scans in our department of radiology from 2016 to 2019. The CT scans were high-resolution abdomen–pelvis CT images with an image section at least from level L1 to the greater trochanter and were acquired due to reasons other than LBP. Exclusion criteria were metastatic and primary malignancy of the musculoskeletal system, previous spinal or pelvic fusion surgery, incomplete image data, and insufficient image quality for software evaluation. Eight-hundred nineteen patients were included. Fifty-two patients had LSTV, six of which had to be excluded due to insufficient image quality. The resulting 46 patients were matched with control patients from the cohort described above, using propensity score matching with a tolerance of 0.01, matching for age, gender, weight category, and indication for CT.

### Image acquisition and radiographic classification

Images were taken by an 80-row or a 320-row CT scanner (Canon Aquillon Prime and Canon Aquillon One Vision, Canon Medical Systems, Otawara, Japan). The chosen isometric slice thickness was 1.0 mm in a medium soft tissue core. CT images were reconstructed with the image visualization and analysis software Amira for Life & Biomedical Sciences certified by the Food and Drug Administration (Thermo Fisher Scientific Materials & Structural Analysis c/o Zuse-Institut Berlin, Germany). Image analysis, muscle segmentation, and measurements were performed by a spine surgeon with experience in measuring radiological spinal parameters, who was trained by a spinal-attending surgeon. LSTV were classified by a radiological resident with specialized training in musculoskeletal radiology (5 years of experience in clinical musculoskeletal imaging and research). A random sample of 50 patients of the original study cohort was additionally classified by an experienced board-certified musculoskeletal radiologist and scored a second time by the primary reader to calculate inter- and intrareader reliabilities.

**Fig. 1:**
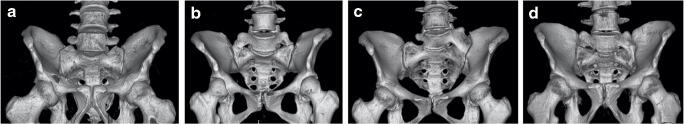
CT scans of different LSTV types in our patient population. **a**. Castellvi Ib, **b**. Castellvi IIb, **c**. Castellvi IIIa, **d**. Castellvi IV. CT = computed tomography, LSTV = lumbosacral transitional vertebrae

In each patient, LSTV were classified according to the Castellvi classification for both the left and the right side as shown in Fig. 1. We then divided all vertebral sides into the following groups: no transition (I), enlarged transverse process (II), pseudarticulation of the transverse process with the sacral bone (III), and fusion of the transverse process with the sacrum (IV).

### Muscle segmentation and measurement

**Fig. 2:**
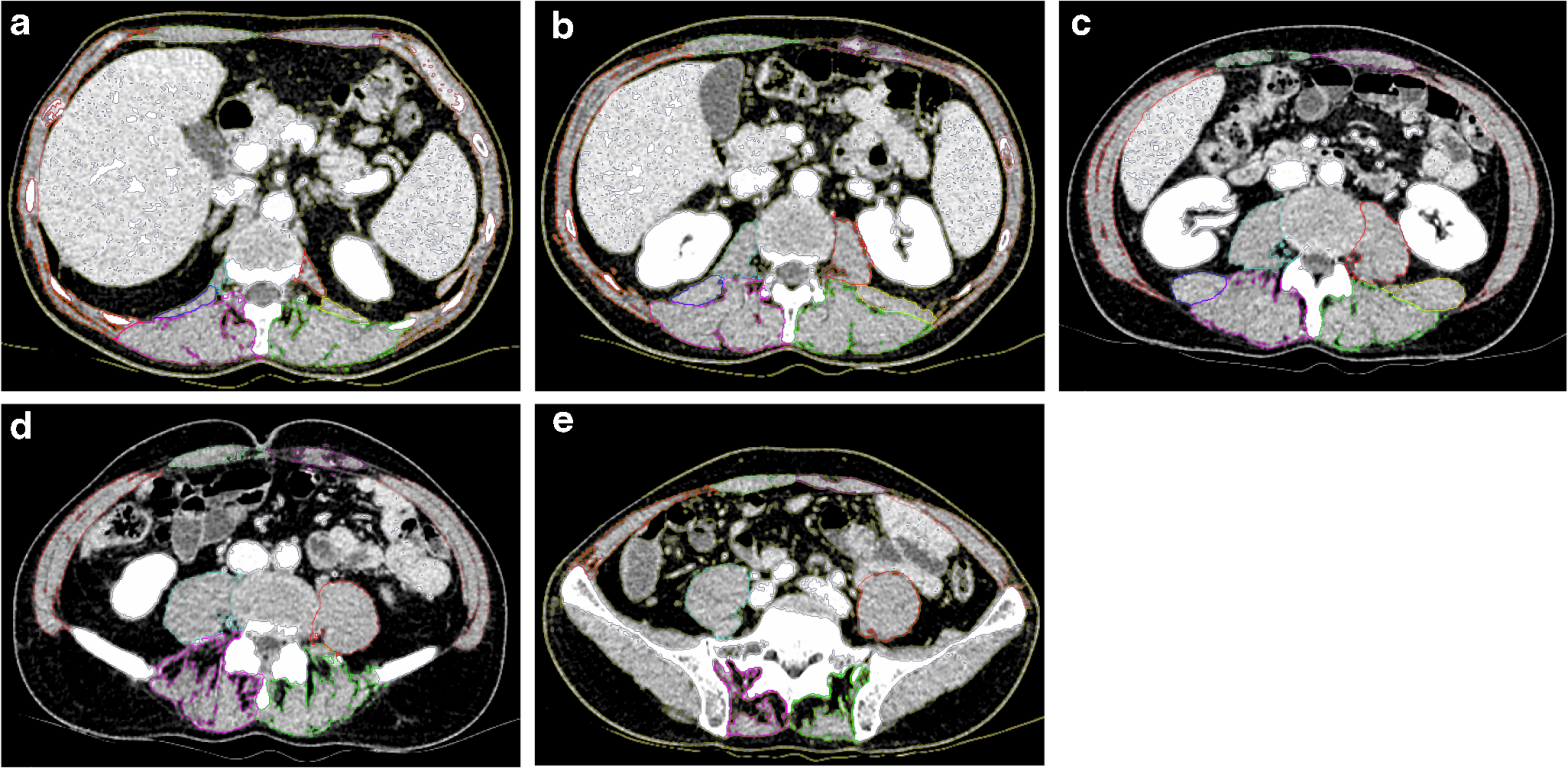
Measurements of the psoas, paraspinal, rectus abdominis, and obliquus abdominis muscles at five levels and the quadratus lumborum muscle at three levels in a patient with Castellvi type IIb. **2a**. Level L1/2, **2b**. level L2/3, **2c**. level L3/4, **2d**. level L4/5, **2e**. level L5/S1

**Fig. 3:**
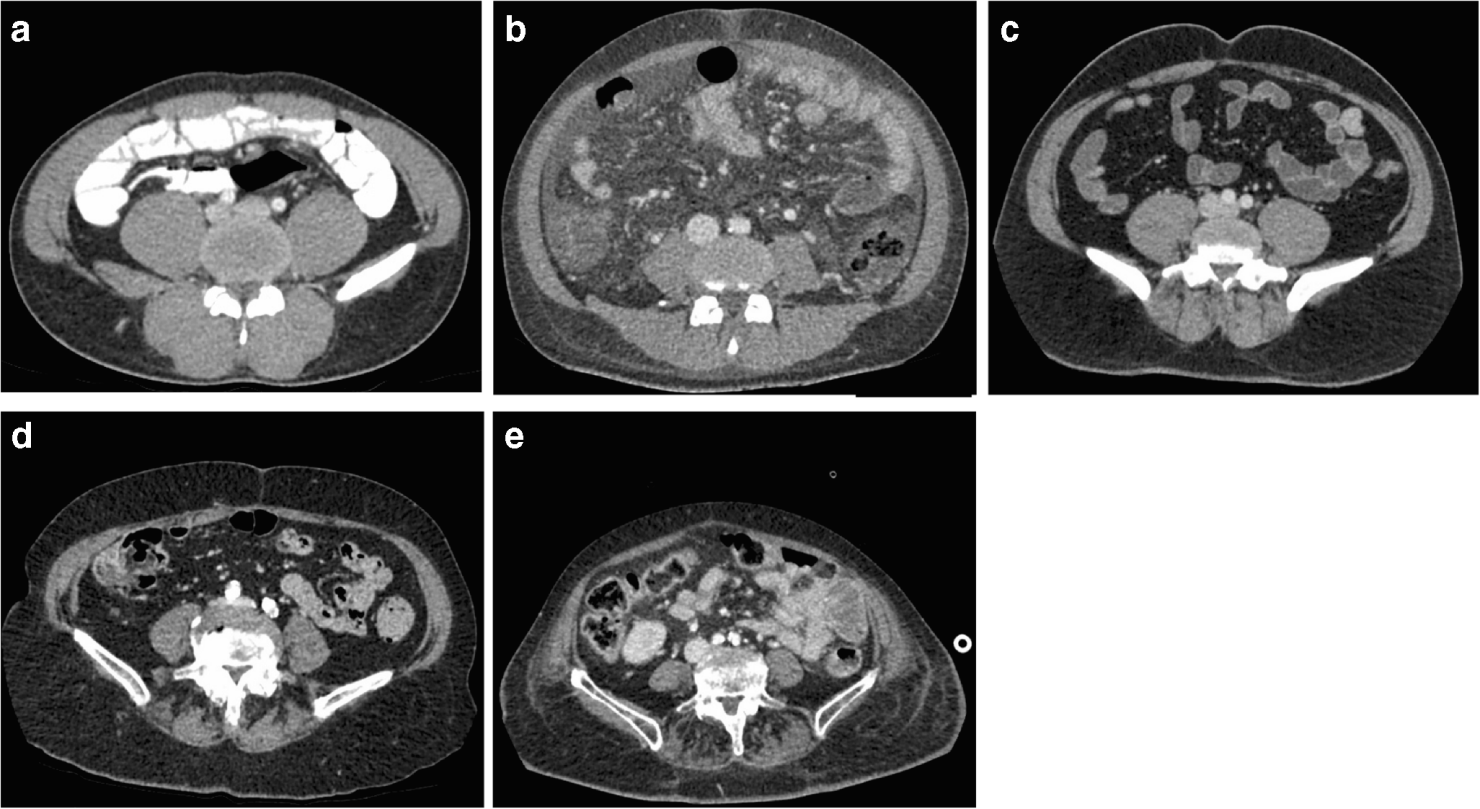
CT scans showing different Goutallier gradings of the paravertebral muscles at level L4/5. **a**. Grade 0: no fatty infiltration. **b**. Grade 1: some fatty streaks. **c**. Grade 2: less than 50% fatty infiltration. **d**. Grade 3: 50% infiltration. **e**. Grade 4: more than 50% fatty infiltration

For image analysis, we used the image processing software Amira. We segmented the muscles from the remaining soft tissue by semi-automated procedures using threshold segmentation tools and manual muscle contouring. We examined muscle cross-sectional area and the muscle volume of the psoas muscle, quadratus lumborum muscle, and paraspinal muscles (erector spinae, multifidus). To verify the accuracy of the measurements, 24 of the 92 patients were remeasured. Twelve patients were randomly selected from both the cohorts of patients with and without LSTV. We differentiated between muscle fibers and fat tissue by using threshold segmentation and assessed the mean muscle density as well as the proportion between muscle mass and fat as signs of muscle degeneration [16]. We determined the muscles’ cross-sectional area in axial sectional imaging at five defined levels. For standardization of our measurements, we determined each measuring level at the center of the anterior edge of the intervertebral disc between the two vertebrae. The uppermost measurement level was between the first and second vertebra below the rib-bearing vertebrae. The other measurement levels were between the second and third, third and fourth, fourth and fifth, and fifth and sixth vertebrae as shown in Fig. 2. Muscle degeneration as expressed by visual fat content was assessed using a classification adapted to the Goutallier grading (Fig. 3): grade 0 shows no fatty infiltration, grade 1 some fatty streaks, grade 2 less than 50% fat, grade 3 50% fat, and grade 4 more than 50% fat [16–18].

### Data analysis

All statistical analyses were performed using SPSS Version 25 (IBM Corporation, New York, USA). Controls were identified from the same study cohort using propensity score matching with a tolerance of 0.01, matching for age, gender, weight category, and indication for CT. The Wilcoxon rank sum test for the comparison of paired samples was used due to the study design as a matched pair analysis. Categorical data were compared using chi-squared test. The associations between the degree of fusion (none, enlarged transverse process, pseudoarticulation, fusion) and muscle size and degeneration were investigated using Spearman’s correlation coefficients. Intra-class correlation coefficients (ICCs) were calculated for inter- and intrareader reliabilities using a two-way mixed model. A significance level of *p* < 0.05 was assumed for all tests.

## Results

### Patients

A total of 46 patients with transitional vertebrae matched with 46 controls. As per study design, LSTV patients did not differ from their matched controls regarding median age (51.5, interquartile range, IQR 37.0–68.0, vs. 50.0, IQR 31.0–70.0; *p* = 0.872), proportion of female patients (23/46 vs. 19/46; *p* = 0.236) or proportion of normal weight patients (29/46 vs. 25/46; *p* = 0.585). CT scans were acquired with query of malignancy (27/46 vs. 25/46), infection (11/46 vs. 20/46), bleeding (2/46 vs. 0/46) or other (7/46 vs. 6/46)—the distribution did not differ significantly between groups (*p* = 0.126). Frequencies of Castellvi grades are shown in Table 1. For classification of LSTV, interreader reliability was good with ICCs of 0.832 (95%CI 0.705–0.904) for the right and 0.712 (95%CI 0.493–0.836) for the left side. Intrareader reliability was very good with ICCs of 0.958 (95%CI 0.925–0.976) for the right and 0.905 (95%CI 0.833–0.946) for the left side.

**Table 1:** Frequencies of Castellvi grades

Type	I	II	III	IV
*n* (%)	14 (30.4%)	22 (47.8%)	6 (13.0%)	4 (8.7%)

### Muscle — volume

**Fig. 4:**
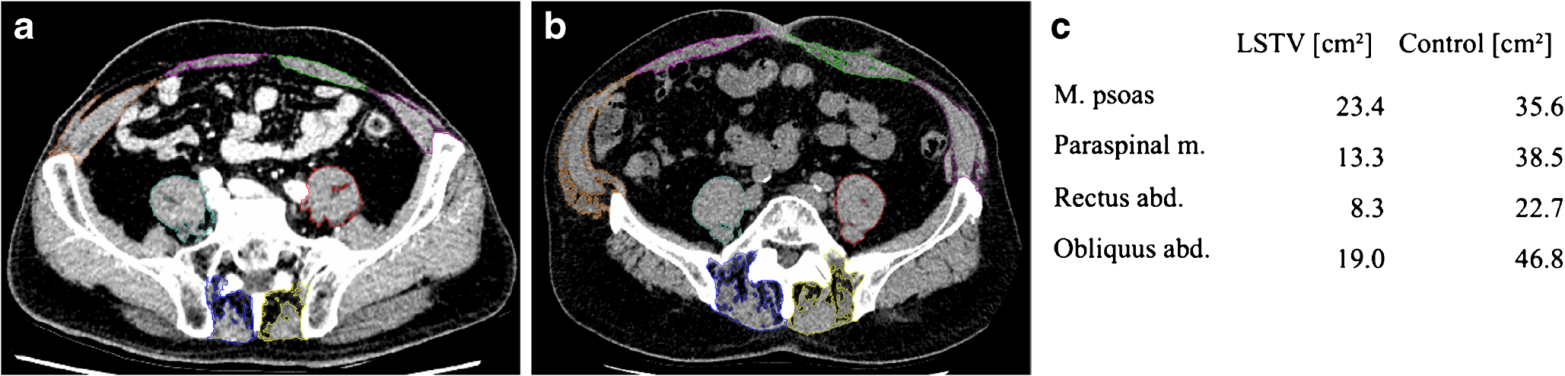
Measurements of the cross-sectional area of the psoas, paraspinal, rectus abdominis and obliquus abdominis muscles at level 5 in a patient with an LSTV Castellvi type IIIb (**a**.) and in a patient without an LSTV (**b**.). **c**. shows the resulting cross-sectional areas

For muscle volume measurements, the intrareader reliability was very good with an ICC of 0.995 (95%CI 0.994–0.996). Total muscle volume was calculated by adding the volumes of all individual muscles of one side. We found a statistically significant (*p* < 0.001) lower total muscle volume for patients with LSTV (mean 707.0 cm^3^, IQR 632.9 cm^3^–1006.4 cm^3^) in relation to our control group (mean 809.7 cm^3^, IQR 689.9 cm^3^–1035.5 cm^3^). Similar findings were found for total muscle volume for M. psoas (*p* = 0.028), paraspinal musculature (*p* < 0.001), M. rectus abdominis (*p* < 0.001), and M. obliquus abdominis (*p* < 0.001). No differences were seen in total muscle volume for M. quadratus lumborum. The cross-sectional area of the individual muscles examined at the five levels is shown in Table 2. An example of muscular atrophy in an LSTV patient is presented in Fig. 4.

**Table 2:** Muscle cross-sectional area in cm^2^ at the five measured levels as well as the muscle volume in cm^3^. Significant values are marked in bold

	M. psoas		M. quad. lumborum		Paraspinal m.		M. rect. abd.		M. obliquus abd.	
	LSTV	Control	LSTV	Control	LSTV	Control	LSTV	Control	LSTV	Control
L1/2	3.15	2.72	2.96	3.01	**21.86**	**22.90**	**5.25**	**5.55**	**23.75**	**24.81**
	*p* = 0.457		*p* = 0.635		***p*** **= 0.015**		***p*** **= 0.005**		***p*** **= 0.027**	
L2/3	6.56	6.87	4.01	4.05	**22.17**	**23.19**	5.07	5.26	**23.37**	**24.69**
	*p* = 0.309		*p* = 0.717		***p*** **= 0.001**		*p* = 0.082		***p*** **= 0.019**	
L3/4	10.21	10.28	5.12	4.93	**21.40**	**22.42**	**5.47**	**5.89**	**23.36**	**24.89**
	*p* = 0.058		*p* = 0.163		***p*** **= 0.003**		***p*** **= 0.003**		***p*** **= 0.003**	
L4/5	**11.79**	**12.24**	–	–	**16.96**	**18.96**	**5.50**	**5.81**	**17.51**	**19.83**
	***p*** **= 0.006**				***p*** **< 0.001**		***p*** **= 0.014**		***p*** **< 0.001**	
L5/S1	9.21	9.33	–	–	**8.14**	**10.07**	**5.61**	**6.29**	**8.55**	**9.59**
	*p* = 0.256				***p*** **< 0.001**		***p*** **= 0.004**		***p*** **= 0.027**	
Volume	121.57	124.95	26.63	27.25	**278.99**	**292.07**	**78.81**	**82.26**	**274.68**	**298.63**
	p = 0.028		*p* = 0.217		***p*** **< 0.001**		***p*** **= 0.001**		***p*** **< 0.001**

### Muscle — symmetry

Asymmetry of muscle mass, defined as the median difference in the cross-sectional area between the left and right side at different levels, did not differ significantly in single levels or in total muscle volume between patients with asymmetrical LSTV (Castellvi Ia, IIa and IIIa; *n* = 31) and controls (*n* = 31).

### Goutallier grading and density

We found significantly more fatty muscle changes for all analyzed muscles in patients with LSTV (M. psoas *p* < 0.04, M. quadratus lumborum *p* < 0.001, paravertebral muscles *p* = 0.011, M. rectus abdominis *p* < 0.001, M. obliquus abdominis *p* < 0.001). Patients with LSTV showed more fatty degeneration at every examined level except the paravertebral muscles at L2/3 and L4/5. Table 3 summarizes the grading by presenting frequencies of modified Goutallier grades. Quantitative analysis of radiodensity as a parameter for fatty degeneration of the muscle as whole did not reveal significant differences between both groups.

**Table 3:** Frequencies of modified Goutallier grades per level. *P* values are derived from chi-square test. Significant values are marked in bold

		LSTV (*n* = 46)				Controls (*n* = 46)				p
Modified Goutallier		I	II	III	IV	I	II	III	IV	
L1/2	M. psoas	89.13%	10.87%	0.00%	0.00%	73.91%	4.35%	0.00%	0.00%	**<0.001**
	M. quad. lumb.	95.65%	4.35%	0.00%	0.00%	70.65%	5.43%	0.00%	0.00%	**<0.001**
	Paraspinal m.	47.83%	52.17%	0.00%	0.00%	60.87%	31.52%	1.09%	0.00%	**0.004**
	M. rectus abd.	61.96%	31.52%	6.52%	0.00%	54.35%	21.74%	2.17%	1.09%	**<0.001**
	M. obliquus abd.	9.78%	88.04%	2.17%	0.00%	35.87%	61.96%	2.17%	0.00%	**<0.001**
L2/3	M. psoas	89.13%	10.87%	0.00%	0.00%	71.74%	8.70%	0.00%	0.00%	**<0.001**
	M. quad. lumb.	86.96%	11.96%	1.09%	0.00%	78.26%	2.17%	0.00%	0.00%	**<0.001**
	Paraspinal m.	48.91%	51.09%	0.00%	0.00%	46.74%	46.74%	2.17%	0.00%	0.101
	M. rectus abd.	51.09%	48.91%	0.00%	0.00%	50.00%	31.52%	0.00%	2.17%	**<0.001**
	M. obliquus abd.	15.22%	84.78%	0.00%	0.00%	30.43%	69.57%	0.00%	0.00%	**0.014**
L3/4	M. psoas	92.39%	7.61%	0.00%	0.00%	80.43%	1.09%	0.00%	0.00%	**<0.001**
	M. quad. lumb.	83.70%	13.04%	3.26%	0.00%	72.83%	3.26%	0.00%	0.00%	**<0.001**
	Paraspinal m.	26.09%	70.65%	3.26%	0.00%	42.39%	51.09%	3.26%	0.00%	**0.024**
	M. rectus abd.	58.70%	40.22%	1.09%	0.00%	57.61%	16.30%	1.09%	4.35%	**<0.001**
	M. obliquus abd.	20.65%	79.35%	0.00%	0.00%	56.52%	40.22%	1.09%	0.00%	**<0.001**
L4/5	M. psoas	90.22%	9.78%	0.00%	0.00%	89.13%	4.35%	0.00%	0.00%	**0.019**
	Paraspinal m.	5.43%	70.65%	23.91%	0.00%	8.70%	61.96%	21.74%	2.17%	0.124
	M. rectus abd.	54.35%	45.65%	0.00%	0.00%	59.78%	27.17%	0.00%	2.17%	**0.001**
	M. obliquus abd.	18.48%	81.52%	0.00%	0.00%	43.48%	56.52%	0.00%	0.00%	**<0.001**
L5/S1	M. psoas	91.30%	8.70%	0.00%	0.00%	93.48%	2.17%	0.00%	0.00%	**0.022**
	Paraspinal m.	0.00%	85.87%	9.78%	4.35%	2.17%	59.78%	35.87%	1.09%	**<0.001**
	M. rectus abd.	59.78%	36.96%	3.26%	0.00%	73.91%	10.87%	0.00%	1.09%	**<0.001**
	M. obliquus abd.	22.83%	77.17%	0.00%	0.00%	45.65%	54.35%	0.00%	0.00%	**0.001**

### Muscle mass and degeneration pending on the degree of expression of LSTV

We found weak but significant negative correlations between the degree of fusion and muscle cross-sectional area for paraspinal muscles (*r* = −0.230; *p* = 0.001), M. rectus abdominis (*r* = −0.211; *p* = 0.002) and M. obliquus abdominis (*r* = −0.2541; *p* < 0.001). In terms of overall muscle volume, we found a significant negative correlation between volume and degree of fusion (*r* = −0.227; *p* = 0.001). Our results show a weak but significant negative correlation between the degree of expression of LSTV and fatty degeneration of M. psoas (*r* = −0.221; *p* = 0.003), M. quadratus lumborum (*r* = −0.381; *p* < 0.001), paraspinal muscles (*r* = −0.320; *p* < 0.001), M. rectus abdominis (*r* = −0.320; *p* < 0.001), and M. obliquus abdominis (*r* = −0.252; *p* = 0.001).

## Discussion

Our results show a significant reduction of total muscle volume in patients with LSTV compared with our control group (707.0 cm^3^ vs. 809.7 cm^3^, *p* < 0.001) as well as a significant reduction of muscle volumes of the paraspinal musculature, M. rectus abdominis, and M. obliquus abdominis. Furthermore, we found significantly more fatty muscle changes in the M. psoas (*p* < 0.04), M. quadratus lumborum (*p* < 0.001), paravertebral muscles (*p* = 0.011), M. rectus abdominis (*p* < 0.001), and M. obliquus abdominis (*p* < 0.001) in patients with LSTV.

As changes in lumbar muscles have been shown to not only affect global sagittal alignment of the spine but also contribute to the development of LBP, we performed this retrospective study to analyze whether there is an association between the occurrence of LSTV and changes in muscle volume, mass, and degeneration. Multiple studies have analyzed the correlation between LSTV and LBP, but evidence on the exact mechanisms of pain development is still lacking.

In patients with LSTV, we found total muscle volume to be significantly reduced. Additionally, paraspinal muscles, psoas muscle as well as both the M. rectus abdominis and M. obliquus abdominis showed significantly lower muscle masses compared to the control group. This reduction of muscle volume and mass in LSTV patients may be caused by restrictions in mobility and a higher degree of connection through osseous fusion [8]. A possible explanation for this may be that biomechanical changes caused by LSTV affect paravertebral muscles as well as abdominal muscles. LSTV therefore is not only a change in bony structure of the spine but may also cause changes in soft tissue anatomy. To the authors’ knowledge, this is the first study showing that with increasingly pronounced disturbances of the lumbo-sacral transition, a significant decrease in the muscle volume of paraspinal and trunk muscles occurs.

Furthermore, we show significantly greater total muscle degeneration of all analyzed muscles in LSTV patients compared to the control group. As mentioned above, morphological changes in LSTV patients lead to altered anatomy and biomechanics with limitation of motion at the same level and hypermobility above the LSTV level [19–21]. This hypermobility of adjacent segments may cause stress on muscular structures, which in turn may lead to increased muscle degeneration. As it has been previously shown that excessive muscular stress caused by decreased muscular activity or repetitive injuries may cause fat infiltration, correct training of stabilizing muscles of the spine may reduce this degenerative process [16, 22, 23]. Regarding the increased fatty infiltration shown in LSTV patients, this may be of particular importance in preventing early muscular degeneration.

Our results also confirm the findings of Bahadir and Ulger [24] showing that an asymmetrical degree of severity of an LSTV does not lead to significant muscular asymmetry. However, patients with LSTV showed significantly less degeneration of the caudal paraspinal muscles and more degeneration of the psoas muscle and the quadratus lumborum muscle. This suggests that patients with LSTV have a different muscular load than patients without LSTV.

As both a reduction in muscle volume and an increase in muscle degeneration may play a role in the pathogenesis of LBP in LSTV patients, preventive strategies against pain development need to be established. There is increasing evidence that in LBP patients, exercise alone or exercise in combination with education programs can reduce the risk of a future episode of LBP as well as future LBP intensity [25, 26]. Additionally, a recent study showed that early physiotherapy significantly improves functionality and reduces pain in patients with chronic LBP [15], highlighting the importance of physiotherapy in the initial treatment of LBP. As our results show changes in volume and degeneration of lumbar and trunk musculature of LSTV patients, which may be caused by or may cause the development of LBP, exercise and physiotherapy need to be considered important strategies in pain prevention. In addition to training autochthonous back muscles, which had a lower volume in patients with LSTV than in the control group, strengthening of the psoas and quadratus lumborum muscles as well as abdominal muscles should be investigated as preventive strategies in LSTV patients.

Some limitations have to be discussed. Due to insufficient data, we excluded six patients, which may have caused an unclear bias. However, as to our knowledge, this still is the largest analysis of CT scans of LSTV patients we are confident the presented data is robust. Due to the retrospective setting, we were not able to include clinical findings such as the occurrence of LBP in our analysis and thus were not able to differentiate between asymptomatic and symptomatic patients. Therefore, we were not able to establish a causal relationship between muscle changes and LBP development in LSTV patients. Additionally, morphological and degenerative changes especially in the facet or disc were not investigated but may play a role in muscle changes and degeneration.

Our results provide evidence that LSTV lead to a reduction of total muscle volume as well as a reduction of muscle volumes of the M. rectus abdominis, M. obliquus abdominis, and paraspinal muscles, all of which showed a significant negative correlation with the degree of fusion. Moreover, we found increased muscle degeneration of all analyzed lumbar and trunk muscles in patients with LSTV, which may be caused by the previously shown limitation of motion at the same level and hypermobility above the LSTV level. To evaluate the impact these changes have on the development of LBP in LSTV patients, further studies including clinical findings such as patient symptoms need to be performed.
